# Trends in bacterial trehalose metabolism and significant nodes of metabolic pathway in the direction of trehalose accumulation

**DOI:** 10.1111/1751-7915.12029

**Published:** 2013-01-10

**Authors:** Rohit Ruhal, Rashmi Kataria, Bijan Choudhury

**Affiliations:** 1 Department of Chemistry, Umeå UniversityUmeå, Sweden; 2 Department of Biotechnology, IIT RoorkeeRoorkee, India; 3 CRANN, Trinity CollegeDublin, Ireland

## Abstract

**Summary:**

The current knowledge of trehalose biosynthesis under stress conditions is incomplete and needs further research. Since trehalose finds industrial and pharmaceutical applications, enhanced accumulation of trehalose in bacteria seems advantageous for commercial production. Moreover, physiological role of trehalose is a key to generate stress resistant bacteria by metabolic engineering. Although trehalose biosynthesis requires few metabolites and enzyme reactions, it appears to have a more complex metabolic regulation. Trehalose biosynthesis in bacteria is known through three pathways – OtsAB, TreYZ and TreS. The interconnections of *in vivo* synthesis of trehalose, glycogen or maltose were most interesting to investigate in recent years. Further, enzymes at different nodes (glucose-6-P, glucose-1-P and NDP-glucose) of metabolic pathways influence enhancement of trehalose accumulation. Most of the study of trehalose biosynthesis was explored in medically significant *Mycobacterium*, research model *Escherichia coli*, industrially applicable *Corynebacterium* and food and probiotic interest *Propionibacterium freudenreichii*. Therefore, the present review dealt with the trehalose metabolism in these bacteria. In addition, an effort was made to recognize how enzymes at different nodes of metabolic pathway can influence trehalose accumulation.

## Introduction

Effect of osmotic stress was studied extensively in bacterial systems. Bacteria accumulate compatible solutes trehalose, glutamate and other osmoprotectants during osmotic stress (Truper and Galinski, 1990). In particular, effects of osmotic stress on *Escherichia coli*, *Corynebacterium* sp. and *Propionibacterium freudenreichii* were reported previously (Strøm and Kaasen, 1993; Carpinelli *et al*., 2006; Cardoso *et al*., 2007; Ruhal and Choudhury, 2012a,b). Altogether, prominent role of trehalose biosynthesis against stress conditions was clearly demonstrated in these bacteria and yeast (Argüelles, [Bibr b1],[Bibr b2]). In yeast, a strong correlation between trehalose content and stress resistance was demonstrated for a variety of stresses such as heat, osmotic stress and ethanol (Hottiger *et al*., 1994; Zancan and Sola-Penna, 2005; Conlin and Nelson, 2007). Similarly, bacteria are subjected to succession of stress conditions during fermentation, which affects their viability and production efficiency. Thus, when the bacterial cell faces stress conditions, dynamic variation arises in the complex metabolic networks (comprise genes, proteins, metabolites, etc.) that subsequently underlie different cellular functions (Belloch *et al*., 2008). Furthermore, the stress response is mediated at the level of transcription, and a number of stress-induced transduction pathways concerning trehalose were reported (Ruis and Schuller, 1995; Estruch, 2000). The research including such molecular responses (like trehalose accumulation) can help us to understand the molecular mechanisms by which cells adapt to fermentation conditions. The advantage of accumulation of compatible solutes is rehydration of bacterial cell (reduction of water activity, restoration of cell volume and turgor pressure) without interfering cellular functions. Trehalose and proline are neutral solutes and hence these are preferred osmoregulator in contrast to potassium ion or glutamate (Csonka and Hanson, 1991). Several studies have reported that trehalose is better as a protein stabilizer than any of compatible solutes because of its unusual ability to alter the water environment surrounding a protein and stabilize the protein in its native conformation (Kaushik and Bhat, 2003; Magazù *et al*., 2005).

Trehalose is a stable non-reducing disaccharide with diverse applications and is widely distributed in nature (Schiraldi *et al*., 2002). Trehalose is accumulated in bacterial cell as a response to stress conditions; hence, it can also be exploited for commercial production of trehalose. The medical use of trehalose in reducing the symptoms of illnesses such as Huntington 's chorea and osteoporosis was reported previously (Higashiyama, 2002; Katsuno *et al*., 2004). Trehalose is found to have nutraceutical value (Hugenholtz *et al*., 2002). It is half as sweet as sucrose, provides sustained energy and elicits a very low insulin response (Higashiyama, 2002; Elbein *et al*., 2003; Kroger *et al*., 2006). Various approaches for the industrial production of trehalose include both enzymatic conversion and its accumulation during the fermentation of glucose using yeast cells (Chi *et al*., 2003). Although enzymatic methods are efficient for trehalose production, bacterial methods are preferable when use of wastes is desirable for commercial production (Li *et al*., 2011). Therefore, in addition to available enzymatic methods, trehalose accumulation was studied for economical production with *Corynebacterium* (Carpinelli *et al*., 2006), *E. coli* (Li *et al*., 2011) and *Propionibacterium freudenreichii* (Ruhal *et al*., 2011; Ruhal and Choudhury, 2012a,b). Knowledge of how exactly trehalose interacts with putative targets and activates metabolic and stress pathways is far from complete and more research must be conducted which in turn could impact industrial microbiology. Furthermore, understanding of trehalose metabolism helps in development of stress-resistant strains used for commercial fermentation processes. The best illustration was shown in a recent research with *Lactococcus lactis*. Interestingly, a recombinant strain was developed with increased accumulation of trehalose and this trehalose-producing strain displayed improved tolerance to acid (pH 3), cold shock (4°C), heat shock (45°C) and against dehydration (Carvalho *et al*., 2011).

As a consequence, in the present review, an effort was made to understand current knowledge of trehalose metabolism together with its physiological significance in bacteria like *E. coli*, *Corynebacterium*, *Mycobacterium* and *Propionibacterium*. Besides, different nodes of metabolic pathways towards trehalose synthesis were also discussed.

## Trehalose metabolism and its significance in *Corynebacterium*, *Mycobacterium*, *E. coli* and *Propionibacterium freudenreichii*

**Figure 1:**
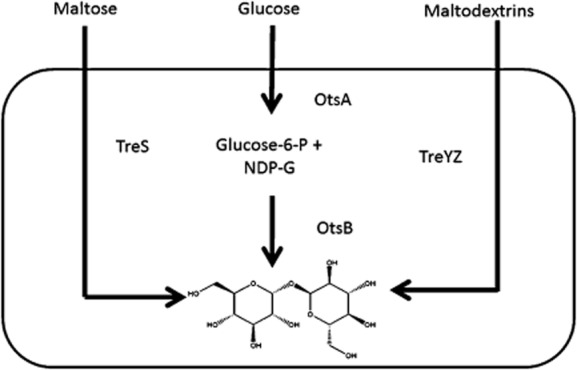
Trehalose metabolic pathways widely distributed in bacteria.

Trehalose biosynthesis was reported in numerous bacteria and exploited for commercial production. In addition, in some bacteria trehalose seems to be trivial for cell physiology. In bacteria three major pathways are known for trehalose synthesis as shown in Fig. 1. The OtsAB pathway, the most common route known to be involved in the stress response of *E. coli* and yeast, proceeds from UDP-glucose and glucose-6-phosphate to form trehalose-6-phosphate, which is subsequently dephosphorylated to yield free trehalose (Fig. 1). The reactions are catalysed by trehalose-6-phosphate synthase (OtsA) and trehalose-6-phosphate phosphatase (OtsB) respectively (De Smet *et al*., 2000). Less-prominent routes for trehalose synthesis are the TreYZ (Kobayashi *et al*., 1996; De Smet *et al*., 2000) and the TreS pathways (De Smet *et al*., 2000; Cardoso *et al*., 2007) (Fig. 1). The substrates of the TreYZ route are oligomaltodextrins or glycogen. In the first reaction step, TreY (maltooligosyl trehalose synthase) transglycosylates a terminal maltosyl residue into a trehalosyl residue before trehalose is liberated through the activity of TreZ (maltooligosyl trehalose hydrolase). Finally, it was described that TreS (trehalose synthase) transforms maltose in a single transglycosylation reaction into trehalose (De Smet *et al*., 2000; Cardoso *et al*., 2007) (Fig. 1).

Further trehalose metabolism and accumulation studies with diverse bacteria including industrially relevant *Corynebacterium*, medically important *Mycobacterium*, research model *E. coli* and food microbe *Propionibacterium freudenreichii* were discussed.

### Corynebacterium glutamicum

*Corynebacterium glutamicum* is an aerobic, Gram-positive soil bacterium most acknowledged for the industrial production of numerous amino acids. It is also used as a model organism for mycolic acid-producing actinomycetes (like *Mycobacterium*). Therefore, study of trehalose production and metabolism seems worth with this bacterium. The defence strategy of *Corynebacterium glutamicum* in osmotic stress was reported in the form of trehalose accumulation and three major pathways were involved with trehalose metabolism – OtsAB, TreYZ and TreS (Wolf *et al*., 2003). Influence of trehalose on *Corynebacterium* physiology was significant as inactivation of trehalose biosynthetic pathway hindered its growth (Tzvetkov *et al*., 2003). Instead, presence of trehalosyl mycolates in the cell wall of this bacterium was predicted through OtsAB and TreYZ pathways (Wolf *et al*., 2003). It was also revealed that during Pi limitation there was high cytoplasmic trehalose content in contrast to maltose (Woo *et al*., 2010). The role of TreS was found to be notable in this bacterium. In general, TreS converts trehalose and maltose reversibly, but TreS was displaying different activities during *in vivo* and *in vitro* conditions. Interestingly, it was observed that TreS was catabolic in nature for substrate trehalose *in vivo* (when cytoplasmic trehalose content was higher) in contrast to *in vitro* conditions (Wolf *et al*., 2003).

Another reserve compound glycogen was reported to play role in osmoadaptation in *Corynebacterium* (Seibold *et al*., 2007), but it was not the only strategy of osmotic adaption in the presence of trehalose as an alternative compatible solute (Seibold *et al*., 2007). In addition, there was a postulation that trehalose metabolism may be related with glycogen accumulation or degradation (Seibold and Eikmann, 2007). Interestingly, *glgX* gene was responsible for glycogen degradation and under osmotic shock it can act as reserve carbon source for conversion into trehalose through TreYZ pathway (Seibold and Eikmann, 2007).

**Figure 2:**
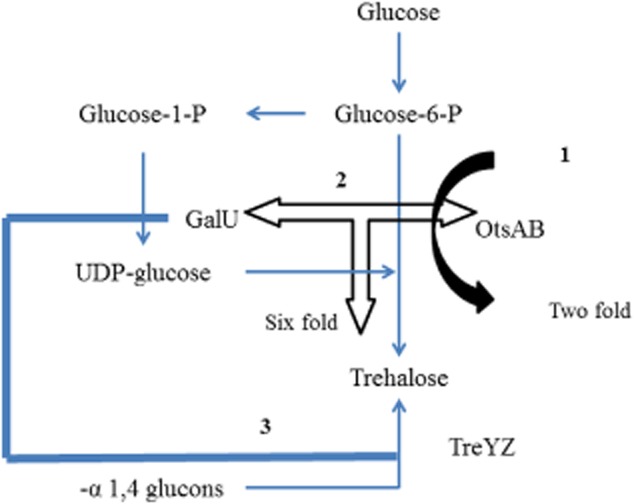
Three strategies of metabolic engineering applied in *Corynebacterium glutamicum* for higher trehalose production. 1 – Overexpression of heterologous *otsAB* from *E. coli* headed twofold trehalose titre (Padilla *et al*., 2004a), 2 – gene *galU*/*otsAB* overexpression led to a sixfold increase in trehalose titre (Padilla *et al*., 2004b) and 3 – gene *galU*/*treYZ* resulted in very high trehalose content (Carpinelli *et al*., 2006).

An effort of higher trehalose production was also reported by Padilla and colleagues by using various metabolic engineering strategies which exploited different combination of genes involved in trehalose biosynthesis. The heterologous expression of *otsAB* (from *E. coli*) in *Corynebacterium* led to twofold increase in trehalose titre (Padilla *et al*., 2004a). Heterologous expression of *otsAB* together with *galU* (UDP-glucose pyrophosphorylase, GalU supplies UDP-glucose) resulted in sixfold increase in trehalose content (Padilla *et al*., 2004b). In another approach of metabolic engineering of different pathways, *galU*/*treYZ* synthetic operon showed significant improvement in trehalose content of up to 7.8 g l^−1^ compared with 1.28 g l^−1^ in wild strain (Carpinelli *et al*., 2006). Thus various approaches for enhancing trehalose accumulation highlighted importance of particular pathway adapted by *Corynebacterium*. The trehalose pathway metabolic engineering strategies adapted and subsequent increase in trehalose content were shown in Fig. 2.

Altogether, important conclusions after study of trehalose accumulation in *Corynebacterium* were – trehalose acts as reserve carbon source, enhanced trehalose accumulation can be achieved through interlinked trehalose biosynthesis pathways and trehalose can be accumulated from glycogen via TreYZ pathway.

### Mycobacterium

It was believed that trehalose acts as reserve energy source or as compatible solute in bacteria but in *Mycobacterium* trehalose was reported as a part of cell wall glycolipids similar to *Corynebacterium* (Brennan and Nikaido, 1995). Since trehalose in *Mycobacterium* has physiological importance, most studies have objective of developing antibacterial drugs targeting trehalose biosynthesis. Trehalose biosynthesis in *Mycobacterium* was reported to be followed through three pathways – OtsAB, TreYZ and TreS (De Smet *et al*., 2000).

**Figure 3:**
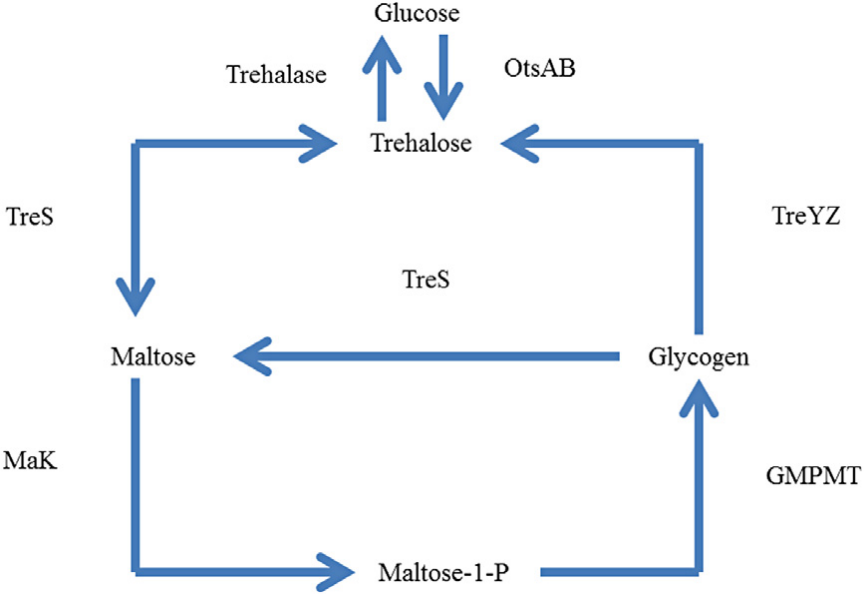
Trehalose metabolism illustrated in *Mycobacterium* in the presence of glycogen and maltose. TreS plays important role when glycogen and maltose present together *in vivo*, but direction of reaction can be determined by concentration of these substrates, role of maltose kinase also presented in the figure. α 1,4 glucan:maltose-1-P maltosyltransferase is shown as GMPMT. Figure adapted from the literature (De Smet *et al*., 2000; Pan *et al*., 2008; Elbein *et al*., 2010; Mendes *et al*., 2010).

One of most interesting trehalose synthesis pathways studied in *Mycobacterium* includes trehalose synthase (TreS). It has been extensively studied as it interconnects *in vivo* synthesis of glycogen and trehalose (Pan *et al*., 2008; Chandra *et al*., 2011). Previously, in a study with *Corynebacterium*, intracellular glycogen was used *in vivo* via TreYZ pathway as a source for trehalose biosynthesis as discussed in above section, but in *Mycobacterium* TreS was reported to be involved in trehalose biosynthesis from glycogen. It was proposed that trehalose synthase, maltokinase and α 1,4 glucan:maltose-1-P maltosyltransferase (GPMT) were involved in synthesis of glycogen when trehalose was present in higher concentration (Elbein *et al*., 2010). Alternatively, role of maltokinase to provide maltose-1-phosphate was considered as metabolic interconnection between trehalose, maltose and glycogen (Mendes *et al*., 2010). Trehalose synthase was purified from *Mycobacterium* and was considered as valuable in synthetic carbohydrate chemistry (Pan *et al*., 2008). Furthermore, isotope exchange studies demonstrated intramolecular mechanism of active site of trehalose synthase and suggested that protein conformational changes were rate-limiting (Zhang *et al*., 2011). The physiological interconnection of these three substrates in *Mycobacterium* has been extensively studied which can be seen altogether in Fig. 3.

Thus from the studies of trehalose metabolism in *Mycobacterium* it can be concluded – trehalose has physiological significance; trehalose, maltose and glycogen may be interlinked through trehalose synthase.

### Escherichia coli

**Figure 4:**
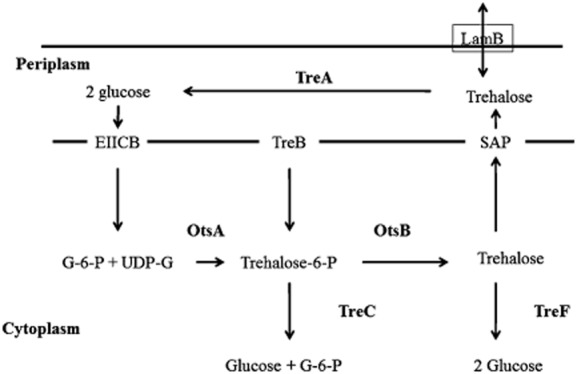
Trehalose metabolism in *E. coli* under hyper- and hypotonic conditions (figure adapted from Purvis *et al*., 2005; Li *et al*., 2011). Detail of enzymes described in text.

Trehalose biosynthesis in *E. coli* was reported through OtsAB pathway (Giaever *et al*., 1988). In addition, trehalose degradation is followed by periplasmic trehalase and amylotrehalase (Strøm and Kaasen, 1993). The best trehalose metabolism in *E. coli* under hypertonic and hypotonic conditions was recently presented as shown in Fig. 4 (adapted from Purvis *et al*., 2005; Li *et al*., 2011). The transmembrane protein LamB can transfer trehalose inside periplasm or help in exit of trehalose from periplasm to extracellular as shown in Fig. 4. Further in periplasm trehalose can be degraded into two molecules of glucose by periplasmic trehalase (TreA) which can be transferred into cytoplasm by phosphotransferase system (EIIAGlc) which channelizes into metabolic pathway where glucose-6-P with UDP-glucose forms trehalose-6-P and subsequently trehalose through OtsAB pathway. Trehalose can be directly transferred into cytoplasm from periplasm by TreB phosphotransport system which is further converted into trehalose-6-P. Trehalose-6-P can be converted into glucose-6-P and glucose by trehalose-6-P hydrolase (TreC) and trehalose can be hydrolysed by cytoplasmic trehalase (TreF) forming glucose which can be channelized into periplasm as shown in Fig. 4 (adapted from Purvis *et al*., 2005; Li *et al*., 2011). Trehalose excretion is helped by stretch-activated proteins (SAP) in the plasma membrane during hyperosmotic conditions. This research group overexpressed OtsAB and used validamycin as trehalase inhibitor and resulted in production of very high trehalose concentration of approximately 1.7 g l^−1^ (NaCl was used as source of osmotic stress) (Li *et al*., 2011).

### Propionibacterium freudenreichii

**Figure 5:**
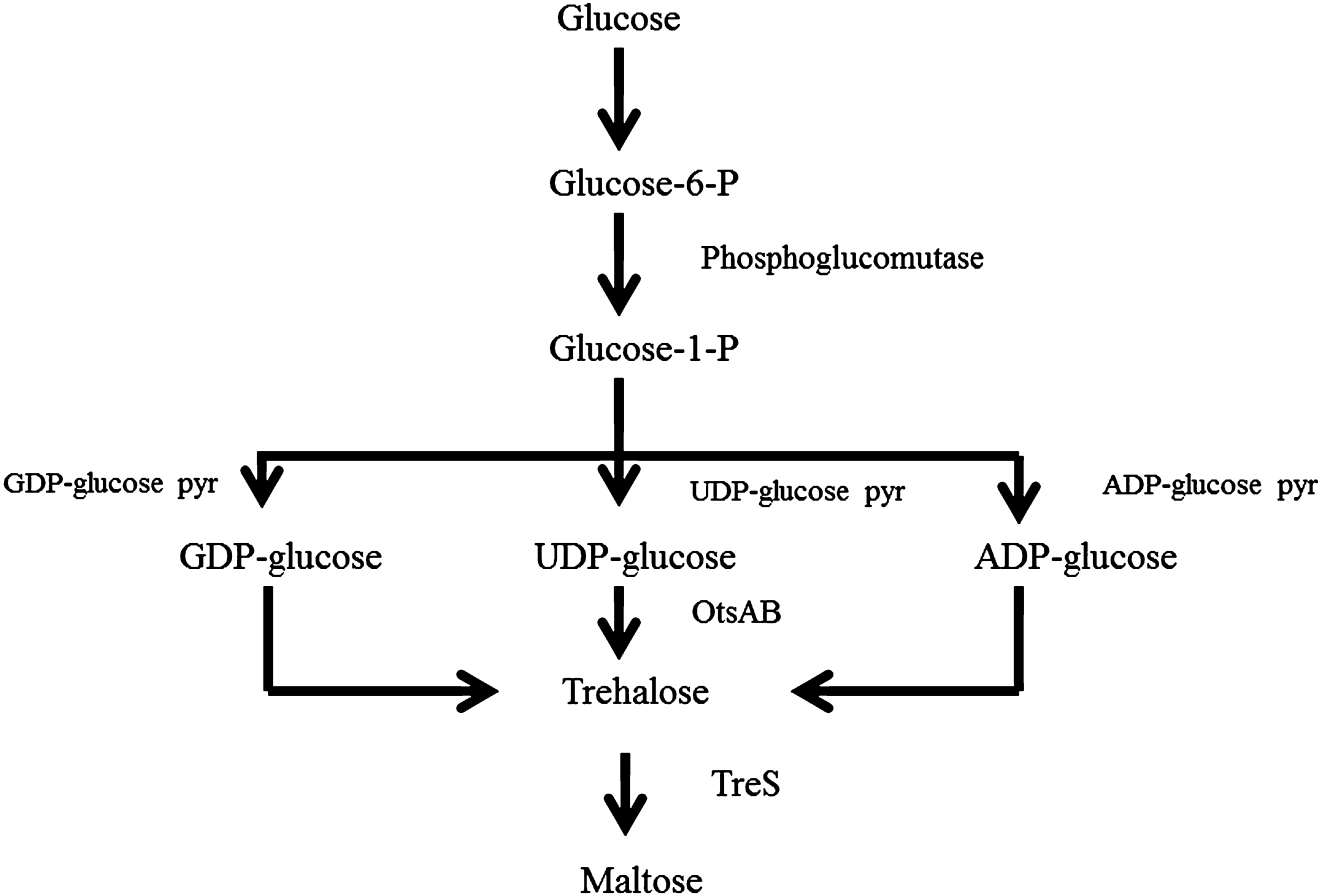
Trehalose metabolism in *Propionibacterium freudenreichii* (Cardoso *et al*., 2007; Ruhal and Choudhury, 2012a).

*Propionibacterium freudenreichii* is a bacterium related with food and probiotic interest (Cousin *et al*., 2010; Hugenschmidt *et al*., [Bibr b28],[Bibr b29]; Thierry *et al*., 2011; Dalmasso *et al*., 2012). It is widely used for cheese ripening. Recently, *Propionibacterium freudenreichii* have been exploited for trehalose production and to understand metabolism. Enhancement of trehalose under different environmental conditions (especially osmotic stress) was reported in *Propionibacterium shermanii* (Cardoso *et al*., 2004). Further biochemical and genetic characterization of trehalose pathways was shown in *Propionibacterium shermanii* under osmotic stress (Cardoso *et al*., 2007). It was proposed that this bacterium follows OtsAB pathway for trehalose synthesis but catabolic pathway is followed through TreS (Cardoso *et al*., 2007). Further gene *otsB* from *P. freudenreichii* was used for developing stress-tolerant strain of *Lactococcus* (Carvalho *et al*., 2011). *Propionibacterium freudenreichii* is widely used as ripening culture in various cheeses and it is found to be metabolically active during storage of cheese at 4°C (Dalmasso *et al*., 2012). Interestingly, in a recent work elevated intracellular trehalose and glycogen accumulation together with slow cell machinery was reported in response to cold temperature which can answer adaptation when cheese is ripened in cold conditions (Dalmasso *et al*., 2012). The concentration of glycogen and trehalose was three and 18-fold higher in cold conditions (4°C) in comparison with 30°C. Genes encoding glycogen synthesis were overexpressed under cold conditions. It was also proposed that there may be interconnection similar to *Mycobacterium* between glycogen and trehalose synthesis since *treS*–*pep2*–*glgE* pathway of glycan synthesis from trehalose was upregulated in cold conditions (Dalmasso *et al*., 2012). Furthermore, *Propionibacterium shermanii* was used for production of higher trehalose yield using crude glycerol (Ruhal *et al*., 2011; Ruhal and Choudhury, 2012a,b). It was also proposed that higher activity of ADP-glucose pyrophosphorylase (ADP-glucose pyr) together with OtsA was responsible for higher trehalose yield (Ruhal and Choudhury, 2012a). The available information on trehalose metabolic pathway is shown in Fig. 5.

## Influence of enzymes at glucose-6-P, glucose-1-P and NDP-glucose nodes for trehalose biosynthesis in central metabolism

**Figure 6:**
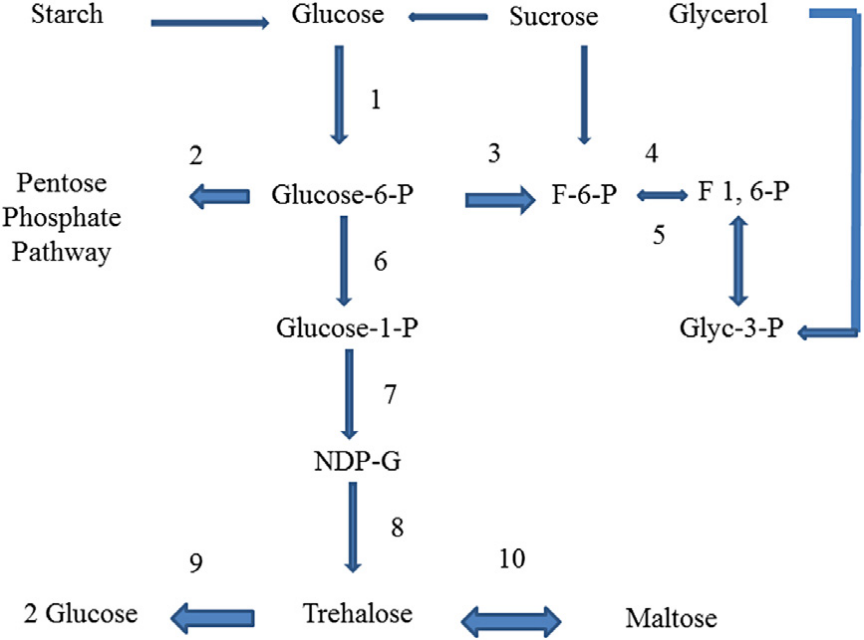
Different nodes of metabolic pathway that can influence trehalose biosynthesis when glycolytic carbon source (glucose, sucrose, starch) and gluconeogenic carbon source (glycerol) used as substrate. 1 – Hexokinase, 2 – glucose-6-P dehydrogenase, 3 – phosphoglucoisomerase, 4 – fructokinase, 5 – fructose 1,6 diP, 6 – phosphoglucomutase, 7 – NDP-glucose pyrophosphorylase, 8 – OtsAB, 9 – trehalase and 10 – TreS.

Monosaccharides (glucose), disaccharides (sucrose), polysaccharides (starch) and gluconeogenic (glycerol) were used for trehalose accumulation. Besides, the carbon channelizes through three important nodes of metabolic pathway – glucose-6-P, glucose-1-P and NDP-glucose as shown in Fig. 6. Since trehalose is synthesized with NDP-glucose and glucose-6-P, these can be significant nodes. Similar to trehalose biosynthesis, microbial production of polysaccharides (like exopolysaccharides, pullulan, glucan) too requires substrate nucleotide sugar (NDP-glucose). Moreover, an insight into these studies explained how enzymes at the different nodes of central metabolic pathway influenced nucleotide sugar synthesis and henceforth exopolysaccharides. Similarly, trehalose biosynthesis may be influenced by enzymes at these nodes. In several previous reports, carbon source influenced synthesis of nucleotide sugars and consequently production of exopolysaccharides, as described by measuring enzyme activities in *Lactobacillus delbrueckii* (Grobben *et al*., 1996) and *Lactobacillus casei* (Mozzi *et al*., 2001). Likewise, exopolysaccharides biosynthesis was correlated to enzyme activities of phosphoglucomutase, epimerase and UDP-glucose pyrophosphorylase in *Streptococcus thermophilus* (Degeest and Vuyst, 2000). Metabolic pathway for β-glucan was proposed in *Pediococcus parvulus* in relation to carbon source by measuring enzyme activities at these three nodes (Velasco *et al*., 2007). This work reported new insight regarding activity of enzymes involved in sugar transport, sugar nucleotide biosynthesis and energy generation. Similarly, metabolic pathway was proposed with the help of enzyme activities in *Lactobacillus helveticus* for exopolysaccharide synthesis which involved enzyme at the branch point of three nodes glucose-6-P, glucose-1-P and UDP-glucose (Torino *et al*., 2005). In fact, in a study with pullulan production in *Aureobasidium*, an analysis of enzyme activities at these nodes and measurement of metabolite UDP-glucose gave some new insight that when more pullulan was synthesized less UDP-glucose was left in cell extract and higher activities of phosphoglucomutase, UDP-glucose pyrophosphorylase and glycosyl transferase were observed in different carbon sources (Duan *et al*., 2008). In conclusion, availability of nucleotide sugars and hence sugar synthesis were correlated to enzyme activities of these three nodes glucose-6-P, glucose-1-P and UDP-glucose which are also affected by numerous environmental conditions including carbon source. Since trehalose biosynthesis involves nucleotide sugar as an important substrate, regulation of these nucleotide sugars through their corresponding enzymes was expected.

Unfortunately, limited metabolic flux studies linked with trehalose biosynthesis were reported. Metabolic flux and elementary mode analysis in *Corynebacterium glutamicum* indicated increased flux towards trehalose formation and it was principle osmolyte in low dilution rate (Rajvanshi and Venkatesh, 2011; Wiitmann and Heinzle, 2002). In addition, carbon flux towards trehalose increased under oxidative stress after deleting transcription repressor McbR (Krömer *et al*., 2008). Thus branches of different nodes of metabolic pathway in relation to trehalose biosynthesis are significant to study. Three important nodes of central metabolic pathway are glucose-6-phosphate (pathway towards glycolysis and pentose phosphate pathway), glucose-1-phosphate and NDP-glucose node (UDP-glucose/ADP-glucose/GDP-glucose) as shown in Fig. 6. Several studies on influences of these various enzymes have been reported previously as discussed above. Hence, it was discussed further how the enzymes at these nodes may have influence on trehalose metabolism as reported in literature.

### Enzymes at glucose-6-phosphate node

#### Glycolysis, pentose phosphate pathway, fructose 1,6 biphosphatase

Although enzymes at branch of glucose-6-P node (Fig. 6) do not have direct control on trehalose metabolism, channelization of carbon (from different carbon source) at these branches can impact trehalose synthesis. This can be more important when carbon source has influence on trehalose synthesis. Effect of carbon source on trehalose biosynthesis was reported in *Propionibacterium freudenreichii*, as higher trehalose accumulated in lactose and lower in lactic acid in comparison with glucose (Cardoso *et al*., 2004). In yeast, it was proposed that the effect of glucose-6-phosphate dehydrogenase was insignificant while in *Corynebacterium* overexpression of glucose-6-phosphate dehydrogenase reduced the trehalose yield (Voit, 2003; Becker *et al*., 2007). This was probably due to reduction of glucose-6-phosphate which is a substrate for trehalose biosynthesis (Becker *et al*., 2007). Similarly, at low osmolality 90 % of flux moves from glucose-6-P node to PPP while it increases towards trehalose synthesis under high osmolality (Varela *et al*., 2004). Alternatively, with gluconeogenic carbon source (like glycerol) fructose 1,6 diPase may be regulatory for trehalose biosynthesis, since carbon enters metabolic pathway through this enzyme. In a similar study with *Propionibacterium freudenreichii*, lactic acid was considered poor for trehalose accumulation and lower activity of gluconeogenic pathway was proposed as probable reason (Cardoso *et al*., 2004). Alternatively, in *Corynebacterium* overexpression of fructose 1,6 biphosphotase led to higher yield of lysine but there was a decrease in trehalose content in the recombinant strain (Becker *et al*., 2005). Thus influence of one enzyme can differ in dissimilar bacteria.

### Enzymes at glucose-1-P node – phosphoglucomutase, NDP-glucose pyrophosphorylase

#### Phosphoglucomutase

Phosphoglucomutase (PGM) converts glucose-6-p to glucose-1-p which is used as substrate for synthesis of NDP-glucose by enzyme NDP-glucose pyrophosphorylase. In yeast, role of phosphoglucomutase for trehalose biosynthesis depends on various other factors like availability of glucose-6-phosphate (Voit, 2003). No direct study of influence of phosphoglucomutase on trehalose biosynthesis in *Corynebacterium* or other bacteria was ever reported.

#### ADP-glucose/UDP-glucose/GDP-glucose pyrophosphorylase

These three enzymes are important for the synthesis of chief substrate known as nucleotide sugars NDP-glucose (ADP-glucose, UDP-glucose and GDP-glucose) for enzyme OtsA. The influence of UDP-glucose pyrophosphorylase (GalU) was studied in *Corynebacterium* and yeast. In fact, heterologous expression of *galU* genes together with *otsAB* from *E. coli* in *Corynebacterium* increased trehalose yield as discussed in above section (Padilla *et al*., 2004a). In yeast during heat shock, 12-fold increase in UDP-glucose pyrophosphorylase resulted in modest increase in trehalose yield (Voit, 2003). Alternatively, NDP-glucose is also involved in various other cellular metabolic activities like synthesis of exopolysaccharides or cell wall formation; thus, to regulate the trehalose biosynthesis individually, cells may adopt NDP-glucose pyrophosphorylase as a controlling element. This fact was further supported in *P. freudenreichii* where it was found that OtsA was more specific to ADP-glucose in crude extract while pure enzyme was more specific to UDP-glucose followed by GDP-glucose and ADP-glucose (Cardoso *et al*., 2007).

### NDP-glucose node – metabolite UDP-glucose, glucose-6-P, glycogen, maltose, trehalase, TreS

#### Concentration of metabolites

The trehalose is synthesized with substrates, UDP-glucose and glucose-6-phosphate; hence, their concentrations and availability can be regulatory in nature. The effect of glucose-6-phosphate on trehalose biosynthesis was predicted insignificant in yeast (Voit, 2003). Likewise, glycogen and maltose accumulation may influence trehalose biosynthesis through TreS and TreYZ pathways (Chandra *et al*., 2011). The unexpected connections of glycogen with trehalose biosynthesis can be reviewed in a recent review (Chandra *et al*., 2011). Similarly, prominent role of TreS in the presence of glycogen and maltose was described in *Mycobacterium* (Pan *et al*., 2008).

#### Trehalase and TreS

Another important enzyme, trehalose synthase (TreS), has important role in trehalose metabolism. This pathway was reported in the complete genome sequence of *P. freudenreichii* (Falentin *et al*., 2010). TreS was a part of catabolic pathway of trehalose in *P. freudenreichii* (Cardoso *et al*., 2007). However, it should be noted that catabolic nature of TreS was reported under osmotic stress. TreS is reversible and can interconvert trehalose and maltose; anabolic nature of this enzyme may be dependent on carbon sources. In bacteria *Pimelobacter* and *Thermus aquaticus* TreS synthesizes trehalose by converting α 1-4 linkage of maltose into α 1-1 linkage (to form trehalose) (De Smet *et al*., 2000). Hence effects of carbon source and osmotic stress can be different for TreS pathway. Role of trehalase has been reported extensively in yeast, *E. coli* and it was shown to be involved in catabolic pathway of trehalose (Argüelles, 2000).

## Conclusions

The solicitous study of trehalose metabolism is important in bacteria, as these can efficiently convert wastes from environment and can make trehalose production economical. It can be concluded that no particular enzyme has total control on trehalose biosynthesis, and similar conclusion was also predicted in yeast where optimum ratios of particular enzymes were found to have influence on trehalose biosynthesis as proposed by Jung and Stephanopoulos (2004). It was also predicted that trehalose metabolism varies in dissimilar bacteria and accordingly depends on requirement of bacterial physiology in the given environmental conditions. Therefore, a more global picture is indeed needed to understand the metabolic regulation of trehalose. Accurate measurement of metabolic fluxes throughout metabolic pathways of bacterial cell by perturbation of carbon source can in some way give better portrait of metabolic status of trehalose biosynthesis.
